# Prevalence and Determinants of COVID-19 Vaccine Acceptance Among Healthcare Workers: A Systematic Review

**DOI:** 10.3389/fpubh.2022.941206

**Published:** 2022-07-28

**Authors:** Belay Desye

**Affiliations:** ^1^Department of Public Health, College of Medicine and Health Sciences, Adigrat University, Adigrat, Ethiopia; ^2^Department of Environmental Health, College of Medicine and Health Sciences, Wollo University, Dessie, Ethiopia

**Keywords:** COVID-19, vaccine, acceptance, healthcare worker, hesitancy

## Abstract

COVID-19 is a major public health problem that has been seriously affecting the global community. Healthcare workers (HCWs) are at high risk of infection due to being directly involved in diagnosing and taking care of patients. Due to this, they were prioritized to receive the initial supply of vaccines. However, vaccine hesitancy has been identified as a major global public health threat. Therefore, this review aimed to synthesize pieces of evidence on the prevalence of COVID-19 vaccine acceptance and determinate factors among HCWs. A systematic search of published articles was identified using PubMed, Science Direct, Web of Science, and Google Scholar for relevant studies of vaccine acceptance and determinant factors among HCWs. Published articles were identified using abstracts and titles of the articles, and articles were assessed for eligibility criteria. The review process was conducted according to the guidelines of Preferred Reporting Items for Systematic Reviews and Meta-Analysis (PRISMA). An electronic database search identified 365 articles, from which 33 full-text articles were included in the systematic review. In this review, the highest rate of vaccine acceptance was reported at 95% and the lowest rate of vaccine acceptance was found at 21%. Factors such as sex (male), age, profession (medical doctors), and previous influenza vaccination were the main positive predictors for COVID-19 vaccine acceptance among HCWs. Concerns about vaccine safety, efficacy, and effectiveness were the main barriers and drivers for vaccine hesitancy. Therefore, to improve the COVID-19 vaccine acceptance among HCWs, governments, public health authorities, and private healthcare systems should work together to provide continuous professional development and training on the safety and effectiveness of the COVID-19 vaccine.

## Introduction

The novel COVID-19 is a global public health threat that has been seriously affecting the international community. COVID-19 was declared a global pandemic in March 2020 after it emerged in Wuhan, China, in November 2019. Globally, as of 21 September 2021, there were nearly 228 million confirmed cases of COVID-19 and over 4.6 million deaths (1).

Globally, governments have imposed several protocols and measures to reduce the spread of COVID-19 like wearing masks, maintaining physical distance, frequent handwashing, and lockdowns. To further prevent and control the spread and deaths associated with COVID-19, vaccines were developed (2). Vaccination is one of the low-cost and most effective measures for the prevention and control of infectious diseases, however, it is challenged by individuals and the general population to accept the vaccine. Due to the variability in COVID-19 vaccines, there are different attitudes and perceptions toward the vaccine (3).

High-risk groups are prioritized to receive the initial supply of vaccines, due to the reason of inadequate supply of COVID-19 vaccines. These high-risk groups are healthcare workers (HCWs) and the elderly, especially those with chronic disease conditions (4). HCWs are at high risk of infection due to being directly involved in diagnosing and taking care of patients (5). Due to their direct or indirect with COVID-19 patients and visitors, HCWs are at high risk of contracting COVID-19 disease (6, 7).

According to WHO, vaccine hesitancy has been identified as one of the major global health threats (8) and the current COVID-19 vaccination is multifactorial, which is due to economic factors and the disease experience of COVID-19, it reduces the acceptance rate (9). Vaccine hesitancy is occurring around the world and an appropriate and effective response to vaccine hesitancy will require information about vaccines that is clear, accessible, and encourage the vaccine (10).

Several studies revealed that not all HCWs were ready to accept the vaccine for COVID-19, there have been increasing reports on hesitancy in receiving the vaccine. For example, a study conducted in Egypt (21%) (11), Ghana (39.3%) (12), and the USA (36%) (13) found that HCWs were intended to accept the COVID-19 vaccine. Most of HCWs reported that their concerns were about the safety and adverse side effects of the vaccines (12–15).

Healthcare workers bridge the gap between healthcare systems and patients. HCWs vaccine acceptance rates correlated with their willingness to recommend the COVID-19 vaccination to their patients. Individuals of the general population who agreed on the benefit of vaccination as recommended by HCWs are reported to have a higher willingness to pay for COVID-19 vaccines (16). People commonly depend on HCWs' information and actions to guide their decisions regarding the acceptance or refusal of the COVID-19 vaccine (17–19). Delays in COVID-19 vaccination among HCWs further prevent herd immunity and will cause an increase in COVID-19-related illnesses and deaths (3, 13).

Attitude and perception of HCWs toward vaccination are major factors that are associated with patient vaccine acceptance and reduced hesitance toward vaccination (20). HCWs intention to use and recommend the vaccine to their patients depends on their knowledge and attitude about the vaccine. Moreover, vaccine hesitancy among the general population has been associated with the level of vaccine hesitancy of HCWs (21). Patients mostly trust and rely on HCWs for vaccine information and vaccine-preventable diseases as well as the public health benefits associated with vaccination (20). It is crucial to consider HCWs' COVID-19 vaccine acceptance to better address the barriers and to widespread vaccination.

Healthcare workers continue to be on the frontline during the current pandemic, countries have prioritized them to be the first to vaccinate (22–24). However, there have been increasing reports on hesitancy in the uptake of vaccines (25). Therefore, examining the acceptance level of the COVID-19 vaccine and determinant factors among HCWs would help policymakers, researchers, and health authorities to design appropriate strategies and interventions to reduce vaccine hesitancy. To my knowledge, this study is the first comprehensive review of COVID-19 vaccine acceptance and determinants among HCWs. This review aimed to provide a synthesis of evidence on the prevalence of COVID-19 vaccine acceptance and determinate factors among HCWs.

## Materials and Methods

### Search Strategies

A comprehensive literature search was conducted. For example, a systematic search of articles was conducted by using the following electronic databases: PubMed, Science Direct, Web of Science, and Google Scholar. The following keywords were used in the search: “COVID-19,” “vaccine,” “acceptance” “healthcare worker,” “hesitancy,” and “willingness.” “AND” and “OR” Boolean operators were employed to integrate the keywords. Moreover, a direct Google search was also conducted. Lastly, the reference lists cited by each eligible article were assessed to identify additional articles. To manage the citation, the Endnote X7 version of the citation manager software was used. This review was conducted following the guidelines of PRISMA (26).

### Eligibility Criteria

**Figure 1:**
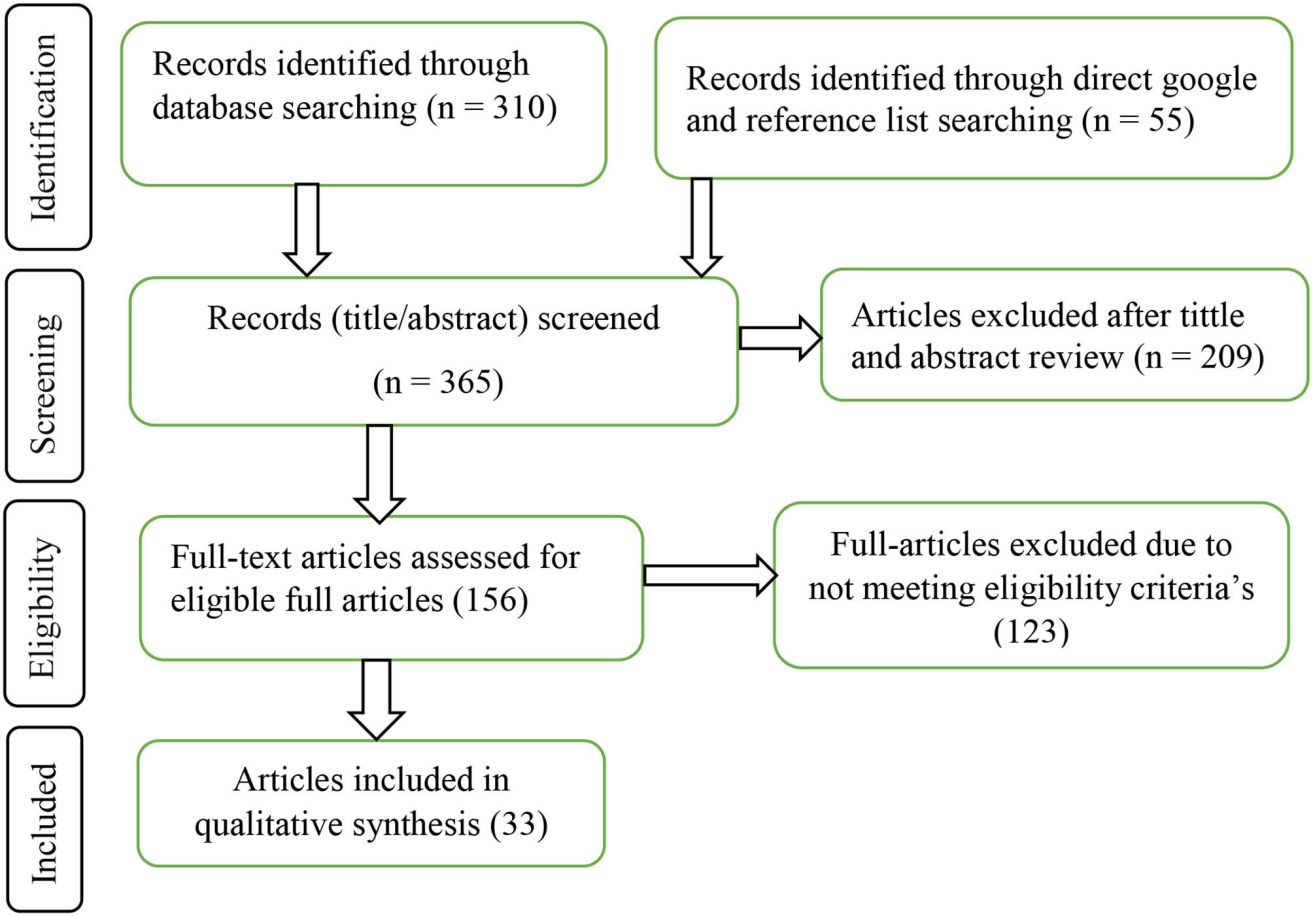
Schematically flow diagram of the article selection process.

In this systematic review, the inclusion criteria were: (1) peer-reviewed published articles, (2) accessed full-text reports, (3) publication language in English, and (4) the major aim of the study was to assess COVID-19 vaccine acceptance or hesitancy and determinants among HCWs, and (5) articles published until 21 September 2021. On the other hand, the exclusion criteria for this systematic review were: articles unable to access the full-text reports after contact with the corresponding authors, as shown in Figure 1.

### Quality Appraisal Method

A total of two reviewers (BD and TT) independently extracted data using the criteria in the data extraction sheet. Inconsistent data between the two reviewers were managed by the involvement of a third reviewer and discussed together. Following data extraction, the findings were organized into three major thematic areas: “acceptance rate,” “determinants of HCWs,” and “main reasons for hesitancy.” Finally, data were presented using text. To ensure consistency, articles were searched systematically using a combination of keywords and Boolean functions by two reviewers independently. The quality of the study was assured using a predefined data extraction form. In addition, the review process was conducted according to the guidelines of PRISMA (26).

## Results

### The Search Processes

A total of 365 articles were identified through the search strategy. Of the total 365 articles, 209 (57.3%) articles were excluded after title and abstract review. Of the remaining 156 articles, 123 (78.8%) articles were excluded due to not meeting the eligibility criteria. Finally, 33 articles were included in the qualitative synthesis (Figure 1).

### Characteristics of the Articles Included in This Review

In the majority of studies, 31 of 33 (94%) of the included full-text articles are research articles, 1 of 33 (3%) are rapid communication, and 1 of 33 (3%) are research letters. 31 of 33 (94%) of the full-text articles are cross-sectional studies, and the remaining 2 of 33 (6%) full-text articles are cross-sectional and qualitative studies. These studies comprised from a total of 25 different countries, studies were done mostly in Africa (*n* = 6), and the USA (*n* = 6), followed by China (*n* = 3) and multinational (*n* = 3). Of the included full-text articles, 10 of 33 (30.3%) are published in 2020, and 23 of 33 (69.7%) are published in 2021.

### COVID-19 Vaccine Acceptance Rate

The results of the COVID-19 vaccine acceptance rates among HCWs in different studies are included in this review are shown (Supplementary Table 1). The highest rate of vaccine acceptance was reported in Asia (a multinational study), which was 95% (27), whereas the lowest rate of vaccine acceptance was found in Egypt, which was 21% (11).

### Determinant Factors of HCWs Toward COVID-19 Vaccine Acceptance

Factors associated with COVID-19 vaccine acceptance among HCWs are presented (Supplementary Table 1. Several demographic characteristics were found to be associated with COVID-19 vaccine acceptance, such as sex (male), age, and profession (medical doctors). Most of the studies indicated that being male HCWs has a positive predictor for COVID-19 vaccine acceptance compared to female HCWs (2, 11, 14, 17, 28–36). Medical doctors were more likely to accept the COVID-19 vaccine than other professions (12, 13, 17, 28, 29, 33, 37–40). Studies also revealed that older age HCWs were more willing to accept the COVID-19 vaccine than younger HCWs (2, 13, 17, 33, 35, 36, 38, 40–44).

Several studies revealed that HCWs who were vaccinated against seasonal influenza in the previous years were more likely to accept the COVID-19 vaccine compared to those not vaccinated (13, 14, 30–34, 38, 43, 45, 46). HCWs having a higher risk of COVID-19 infection, such as encountering suspected or confirmed patients with COVID-19, and had a higher willingness to be vaccinated against COVID-19 (2, 14, 29, 43). In addition, factors such as perceived susceptibility, perceived severity, perceived benefit of taking action, perceived barriers, and cues to action were associated with COVID-19 vaccine acceptance (2, 33, 38, 47).

Most studies indicated that HCWs encourage their family, friends, and patients to take the COVID-19 vaccine (11, 13, 37, 42, 45). Regarding receiving a safe and effective vaccine, a majority of studies revealed that HCWs agreed to receive a safe and effective vaccine (11, 37, 42, 45, 48). Findings revealed that 93.1% in Libya (42) and 77.4% in China (37), HCWs reported that the COVID-19 vaccine should be provided free of charge, while only 48.2% would purchase it if not available for free (42). However, 71.6% of HCWs believed that would be a difficulty in proper and equitable vaccine distribution (42).

According to Elhadi et al. (42) and Luoda et al. (37) reported that most of HCWs agreed that the COVID-19 vaccine could prevent and reduce the COVID-19 burden. Around 48% of HCWs reported that the administration of COVID-19 vaccination should be on a voluntary basis (13). According to Fares et al. (11), 51.7% of HCWs reported that being vaccinated helped to build immunity. The perceptions of HCWs were significantly different among sex, income, academic level, willingness status, and perception level (29).

### Reasons of HCWs for COVID-19 Vaccine Hesitance

Several studies reported that the main concern of HCWs toward COVID-19 vaccine acceptance was the safety of the vaccine. Concern of vaccine safety included potential side effects (2, 11–14, 17, 29–31, 34, 35, 37, 38, 40–42, 45, 48–51). Vaccine efficacy and effectiveness were also the major reasons of HCWs for COVID-19 vaccine hesitancy (13, 14, 17, 30, 38, 42, 51). HCWs distrusted the government, the health authorities, and the way the vaccine was developed rapidly, causing them to be concerned that the vaccine may have safety issues (17, 35, 39–41, 44, 51).

## Discussion

This review examined the acceptance level of HCWs for COVID-19 vaccination and identified determinant factors. The COVID-19 vaccine acceptance is a major concern globally. HCWs' attitudes and perceptions toward the COVID-19 vaccine play a crucial role in the general population. Due to misinformation, health beliefs, and conspiracy theories, HCWs may develop vaccine hesitancy, which can influence their decision to receive the vaccine and may not promote the vaccine to others (28).

The findings revealed that in this review, there was a higher acceptance rate (95%) in Asia (27) and a lower acceptance rate (21%) in Egypt (11) for receiving the COVID-19 vaccine if available. The different findings might be because vaccine acceptance may vary over time as additional information was provided about the safety and risk of the vaccine (36). The main purpose of HCWs accepting the COVID-19 vaccine was to protect themselves and others from COVID-19 infection. To achieve a high vaccine acceptance among HCWs and the general population, governments and health authorities should address the concerns about the safety and adverse side effects of the COVID-19 vaccine as early as possible. In addition, governments and health authorities should strengthen public education in both traditional and social media on accurate and sufficient information regarding COVID-19 and the advantage of accepting COVID-19 vaccines (11, 12, 52).

According to Kabamba Nzaji et al. (28) and Garrett (53), they explained that the major barriers to low willingness to vaccinate against COVID-19 vaccine could be due to multiple rumors, conspiracy theories, health beliefs, and the spread of misinformation on the safety of vaccines through social media. The low acceptance of the COVID-19 vaccine among HCWs may have a negative impact on patients and the general population (13). Providing accurate information on social media with credible sources will help to avoid this misinformation. Therefore, public health strategies are urgently needed to address the barriers contributing to the low acceptance of the COVID-19 vaccine.

Medical doctors were more likely to accept the COVID-19 vaccine if available compared to other professions (pharmacy, medical laboratory, and nurses) (12, 13, 28, 37). HCWs who have higher academic degrees and are directly involved in patient care were having a higher willingness to accept the vaccine (32, 36, 37, 50). Most HCWs who perceived themselves to be at risk of COVID-19 infection, prior to COVID-19 exposure, and perceived that the disease is more severe were more willing to accept the vaccine than those who are not at risk and considered the disease as mild (13, 54). Chew et al. (27), reported that HCWs who were willing to vaccinate were more likely to have the perception that the pandemic was severe and the vaccine was safe and effective.

The findings reported that male HCWs were significantly higher to accept the COVID-19 vaccine if available, compared to female HCWs (11–13, 28). This might be due to the higher infection rate of COVID-19 among male HCWs (30). This might be also due to risk perception toward the COVID-19 disease being higher in men compared to women. Moreover, people most likely at risk for COVID-19 infection were more willing to accept the vaccine (28).

Grech et al. (55) and Detoc et al. (56) supported that there is a higher acceptance of the COVID-19 vaccine among older HCWs than younger HCWs as they are more vulnerable to the COVID-19 disease. The findings might be due to the increased risk of infection associated with age. Age is a known risk factor for COVID-19, and comorbidity is common in older adults, which may lead them to a greater risk of the COVID-19 infection. Older people were also associated with higher rates of COVID-19 mortality as compared with younger people. Therefore, older HCWs are more willing to be vaccinated because they are at high risk. HCWs with chronic disease had higher vaccination acceptance because of comorbidities associated with the severity and poor prognosis of COVID-19, which can increase the risk of death (41).

Previous influenza vaccination history were important predictors for taking COVID-19 vaccination (13, 14, 30–34, 38, 43, 45, 46). It suggested a possible correlation between vaccine acceptance and behavior among different vaccines (13, 14, 34, 45). This also may indicate that taking the vaccine could be a habit, which could help to increase the willingness for the COVID-19 vaccine (14). Smith et al. (57), reported that effectiveness of the vaccine, previous vaccinated history and susceptible to infections are factors that can affect the acceptance of childhood influenza vaccines.

Healthcare workers who have a family member or friend infected with COVID-19 were more likely to accept the COVID-19 vaccine compared to those who had not. This might be due to HCWs having gained more knowledge about COVID-19 and its effects on human health, they may protect themselves if the COVID-19 vaccines become available (12, 42).

This review identified that the main reasons or drivers of HCWs for the hesitancy toward the COVID-19 vaccine were the concerns about safety, efficacy, and effectiveness of the vaccine. These findings are supported by (11–13, 15, 17, 29, 38, 45, 58). WHO states that there are rigorous and vigorous testing trials in place to grant that approved COVID-19 vaccines are safe and effective (1). Individuals who perceive vaccines are safe and effective are more likely willing to accept vaccination (59, 60). Insufficient data and knowledge about the COVID-19 vaccine increased vaccine hesitancy (44, 61). Understanding and addressing HCWs vaccination barriers are important to improve the COVID-19 vaccine acceptance rate and can provide a crucial lesson for other infectious disease crises. They need more information regarding the vaccine safety before being vaccinated, and tailored communication strategies are also needed to disseminate more to improve HCWs' confidence toward COVID-19 vaccination, and to increase their acceptance rate (13, 17, 44, 51).

### Limitations of the Review

The review included articles written only in the English language and included limited articles.

## Conclusion

In this review, the large variability of the COVID-19 vaccine acceptance rate was found in different countries and regions of the world. Sex (male), age, profession (medical doctors), and previous influenza vaccination history were positive predictors for COVID-19 vaccine acceptance among HCWs. Concerns about vaccine safety, efficacy, effectiveness, and distrust within the government and the rapid development of the vaccine were the main barriers and drivers for vaccine hesitancy. Multiple rumors, various misconceptions, health beliefs, conspiracy theories, and concerns about the safety and effectiveness of the vaccine need to be addressed to reduce the hesitancy of the vaccine. Moreover, understanding socio-demographic characteristics and determinants may help to reduce the hesitancy of the vaccine. Therefore, to improve the COVID-19 vaccine acceptance among HCWs, governments, public health authorities, and private healthcare systems should work together to provide continuous professional development and training on the safety and effectiveness of the COVID-19 vaccine.

## Data Availability Statement

The original contributions presented in the study are included in the article/Supplementary Material, further inquiries can be directed to the corresponding author.

## Author Contributions

The author made a significant contribution to the conception and design, acquisition of data, and interpretation of data, critically reviewed the article, gave final approval of the version to be published, and agreed to be accountable for all aspects of the work.

## Conflict of Interest

The author declares that the research was conducted in the absence of any commercial or financial relationships that could be construed as a potential conflict of interest.

## Publisher's Note

All claims expressed in this article are solely those of the authors and do not necessarily represent those of their affiliated organizations, or those of the publisher, the editors and the reviewers. Any product that may be evaluated in this article, or claim that may be made by its manufacturer, is not guaranteed or endorsed by the publisher.
