# Supplementary material for: Prevalence and Determinants of COVID-19 Vaccine Acceptance Among Healthcare Workers: A Systematic Review
Source: Front Public Health. 2022 Jul 28;10:941206. doi: 10.3389/fpubh.2022.941206 (PMC9366855; doi:10.3389/fpubh.2022.941206)
Supplement: Supplementary file 1 [file Appendix.docx]

| S.N | Author | Country/Region | Type of research | Study design | Acceptance  rate % | Determinant factors | Reasons for vaccine hesitancy |
| --- | --- | --- | --- | --- | --- | --- | --- |
| 1. | Agyekum et al.,  2021 (12) | Ghana | Research article | CS | 39.3% | Sex (female), profession (medical doctors), do not know if their relatives had been diagnosed with COVID-19, and the accuracy of measures taken by the government to fight COVID-19 | Vaccine safety, and adverse side effects |
| 2. | Fares et al., 2021 (11) | Egypt | Research article | CS | 21% | Sex (male), encountering directly with COVID-19 patients, recommending COVID-19 vaccination for others, getting nonobligatory vaccinations, hospital or medical center advice to get the vaccine, vaccine producers are interested primarily in health, trusting the pharmaceuticals for a safe and effective vaccine, authorities discuss information about side effects openly, and heard of bad reaction related to COVID-19 vaccination | Inadequate clinical trials and concern of the safety of the vaccine |
| 3. | Luodan et al., 2020 (28) | China | Research article | CS | 67.1% | Vaccine safety and effectiveness, COVID-19 can be prevented by vaccination, free of charge COVID-19 vaccine, COVID-19 vaccine approved for license, higher academic degree and professional rank and titles | Safety of the vaccine, and vaccine charge |
| 4. | Elharake  et al.,  2021(29) | Saudi  Arabia | Research  Article | CS | 64.9 % | Age (≥18), sex (female), country of origin  , education (≥ high school), chronic  illness (yes), and know someone who  has been sick with COVID-19 (No) | fear of potential  side effects and  lack of trust on  vaccine |

Table 1. Summary of full-text articles which reported the vaccine acceptance rate, determinant factor, and reasons for vaccine hesitancy among HCWs.

N.B: CS = Cross-sectional study

Table 1. (Continued)

| **5.** | Verger  et al., 2020 (30) | France, Belgium, and Canada | Rapid communication | CS | 71.6% | Vaccine safety and effectiveness, and being vaccinated against seasonal influenza | Vaccine safety |
| --- | --- | --- | --- | --- | --- | --- | --- |
| **6.** | Chew  et al., 2021 (27) | Asia | Research article | CS | 95% | Perceived pandemic and vaccine harm | Perceived susceptibility to the pandemic and a pro-socialness mindset |
| **7.** | Nzaji  et al., 2020 (31) | Congo | Research article | CS | 27.7% | Sex (male), profession (medical doctors), and having a positive attitude toward COVID-19 | Vaccine quality and false information’s |
| **8.** | Paudel  et al., 2021 (32) | Nepal | Research article | CS | 38.3% | Sex (male), educational status, profession (medical practitioners ), and HCWs involved in patient care | Vaccine safety |
| **9.** | Elhadi  et al., 2021 (33) | Libya | Research article | CS | 79.6% | Age (>31), currently infected with COVID-19, have a family member or friend infected with COVID-19, and have a family member or friend died due to COVID-19 | Vaccine related complications and vaccine efficacy |
| **10.** | Temsah et al., 2021 (34) | Saudi Arabia | Research article | CS | 45.3% | Vaccine efficacy, preferences and vaccine manufacture country | Vaccine safety and adverse side effects |
| **11.** | Spinewine et al., 2021 (35) | Belgium | Research article | CS | 62.9% | Age (>44), sex (female), profession (medical doctors, nurses or nurse assistants), seasonal influenza vaccination, perceived benefits of taking action and cues to action | Vaccine safety and effectiveness |
| **12**. | Littig et al., 2021(36) | Germany | Research article | CS | 91.7% | Age and profession (residents) | Lack of trust in authority and pharmaceutical companies |

N.B: CS = Cross-sectional study

Table 1. (Continued)

| **13.** | Youssef  et al.,  2021 (37) | Lebanese | Research article | CS | 58% | Sex (male), frontline worker, receiving influenza vaccine, rural area, history of COVID-19, novelty of vaccine, side effect, reliability manufacture, vaccine frequency, and vaccine availability and accessibility | Vaccine safety and efficacy, and novelty of the vaccine |
| --- | --- | --- | --- | --- | --- | --- | --- |
| **14.** | Parente  et al.,  2021 (38) | Kansas  City | Research  article | CS | 59.5% | Sex (male), previous influenza  vaccination, increased concern about  COVID-19, and education level  (post-graduation) | Vaccine safety,  efficacy, long-term  side effects, cost and  risk to benefit ratio |
| **15.** | Alle and Oumer, 2021 (39) | Ethiopia | Research article | CS | 42.3% | Age (>30 years), profession (physician, pharmacy and laboratory) | Adverse side effects, vaccine effectiveness and lack of trust on vaccine development |
| **16.** | Shekhar et al., 2021 (13) | USA | Research article | CS | 36% | Age(>45), sex (female), profession (medical doctor and nurse or nurse assistance), being vaccinated against seasonal flu | Vaccine safety and effectiveness |
| **17.** | Meyer et al, 2021 (40) | not specified | Research Letter | CS | 55.3% | Time for completing survey and patient facing employees | Insufficient data, adverse side effect and lack of trust on vaccine development |
| **18.** | Al-Sanafi and Sallam, 2021(41) | Kuwait | Research article | CS | 83.3% | Sex (female), profession (nurse and laboratory worker), HCWs in private sector, non-Kuwait citizenship, and low educational level are significantly associated with vaccine hesitancy | COVID-19 vaccine conspiracy beliefs |
| **19.** | Kwok et al., 2020 (42) | China | Research article | CS | 63% | More confidence, younger age, less complacency , and more collective responsibility | Vaccine effectiveness, side effects, and effective duration |

N.B: CS = Cross-sectional study

Table 1. (Continued)

| **20.** | Shaw  et al., 2021 (17) | USA | Research article | CS+ Qua | 57.5% | Physicians and research scientists, age, sex (male), Caucasian and Asian | Vaccine safety, side effect, efficacy, and lack of trust on vaccine development |
| --- | --- | --- | --- | --- | --- | --- | --- |
| **21.** | Huynh et al., 2021(43) | Vietnam | Research article | CS | 76.1% | Perceived benefits of vaccination, perceived barrier, perceived susceptibility and severity of COVID-19, and cues to action | Not mentioned |
| **22.** | Kanyike et al., 2021(44) | Uganda | Research article | CS | 37.3% | Marital status (single), sex (male), perceived risk of getting COVID-19 in the future, receiving any vaccine in the past 5 years, and COVID-19 vaccine hesitancy | Not mentioned |
| **23.** | Malik et al.,  2020 (45) | Pakistan | Research article | CS | 70.25% | Sex, age, taking direct of COVID-19 patients, higher education, and prior COVID-19 infection | Vaccine safety, effectiveness, and prior COVID-19 exposure |
| **24.** | Wang et al.,  2020 (14) | China | Research article | CS | 40% | Sex (male), having chronic conditions, HCWS in the private sector, previous influenza vaccination, and encountering with suspected /confirmed COVID-19 patients | Vaccine efficacy, effectiveness and safety,  believing it is unnecessary, and no time to take it |
| **25.** | Lucia et al.,  2020 (46) | USA | Research article | CS | 77% | Vaccine side effects | Vaccine side effects, safety and efficacy, distrusting the information from public health expert, rapid development of vaccine, politicized |
| **26.** | Gagneux-Brunon  et al., 2020 (47) | France | Research article | CS | 76.9% | age, sex (male), physicians, fear about COVID-19, perceived individual risk and previous influenza vaccination | Not mentioned |

N.B: CS = Cross-sectional study, Qua = Qualitative studies

Table 1. (Continued)

| **27.** | Manning et al., 2021 (2) | USA | Research article | CS+  Qua | 60% | Faculty, age ≥60, sex (male), providing direct patient care, whether their colleagues acquired COVID-19, and perceived impact on their own health | Vaccine safety and side effects |
| --- | --- | --- | --- | --- | --- | --- | --- |
| **28.** | Qunaibi et al, 2021 (48) | Multinational | Research articles | CS | 70 % | Age (>30), gender (female), suspected or confirmed COVID-19, vaccine type and influenza vaccine | Side effects and vaccine safety |
| **29.** | Kose et al, 2020 (49) | Turkey | Research article | CS | 68.6% | Sex (male), students, younger age group, being received previous influenza vaccination | Vaccine effectiveness and side effects, trusting their own immune system, be protected from the disease, and not afraid of getting sick |
| **30.** | Gadoth  et al, 2020 (50) | USA | Research article | CS | 33.5% | Fast-tracking the development timeline, novelty and unfolding science of SARS-CoV-2, political climate, age (≥50), prescribing clinicians, and sex (male) | Concerns about fast-tracking regulatory procedures, and a lack of transparency and/or publicly available information on newly developed vaccines |
| **31.** | Unroe et al, 2021 (51) | USA | Research article | CS | 69% | Age (>60years), sex (male), and race | Vaccine side effects, and effectiveness, religious issues, too new, a lack of trust, need for more research and politicized |
| **32.** | Dzieciolowska  et al, 2021 (52) | Canada | Research article | CS | 80.9% | Sex (male), age (>50 years), rehabilitation center workers, and occupational COVID-19 exposure | Novelty of the vaccine, wanting others to receive it first, and lack of time for decision-making. |
| **33.** | Fakonti  et al,  2021 (53) | Cyprus | Research  article | CS | 30% | Sex (female), received influenza  vaccine, received vaccines suggested  by HCWs, and private hospital | Fear of side effects, and  concerns about the vaccine’s  expedited development |

N.B: CS = Cross-sectional study, Qua = Qualitative studies
